# Supplementary material for: Exploring concurrent validity of the CLN2 Clinical Rating Scale: Comparison to PedsQL using cerliponase alfa clinical trial data
Source: PLoS One. 2024 May 22;19(5):e0302382. doi: 10.1371/journal.pone.0302382 (PMC11111014; doi:10.1371/journal.pone.0302382)
Supplement: S1 File — (DOCX) [file pone.0302382.s001.docx]

**SUPPORTING** **INFORMATION**

**Statistical Limitations**

The large number of tests used in this study also meant that a number of standard assumptions associated with regression analyses were not thoroughly tested. Standard assumptions included a linear relationship between variables of interest, independence and homoscedasticity. However, visual inspection of residual plots across all models indicated no violation of these assumptions (results not shown). An additional assumption for all the regression analyses that were performed was that the relationship between the independent and dependent variable does not change over time (study week). When acting as dependent variables, Clinical Rating Scale (Total score) and PedsQL (Total and domain scores), were treated as continuous variables.

For multicollinearity analyses, it was assumed that any multicollinearity between the domains remained constant over time and normality was assumed. It must also be noted that for the multicollinearity analyses, which used data where multiple observations were taken on a single patient, the independent errors assumption was violated. Furthermore, due to there being only one score of zero in the vision domain for Study 201/202, the vision domain was highly correlated with the intercept in the regression analyses giving uninformative large condition indices. Hence, condition indices for models without the intercept were calculated when the vision domain was included in the analysis.
